# Supplementary material for: Lipase Addition Promoted the Growth of Proteus and the Formation of Volatile Compounds in Suanzhayu, a Traditional Fermented Fish Product
Source: Foods. 2021 Oct 21;10(11):2529. doi: 10.3390/foods10112529 (PMC8625596; doi:10.3390/foods10112529)
Supplement: Supplementary file 1 [file foods-10-02529-s001.zip › foods-1394574-supplementary.pdf]

Supplementary data

Lipase addition promoted the growth of *Proteus* and the formation of volatile compounds in *Suanzhayu*, a traditional fermented fish product

Cuicui Jiang [a], Mengyang Liu[a], Xu Yan [a], Ruiqi Bao[a], Aoxue Liu[a], Wenqing Wang[a], Zuoli Zhang[a], Huipeng Liang [a], Chaofan Ji[a], Sufang Zhang[a], Xinping Lin [a,*]

[a] *National Engineering Research Center of Seafood, Collaborative Innovation Center of Provincial and ministerial co-construction for Seafood Deep Processing, Liaoning Province Collaborative Innovation Center for Marine Food Deep Processing, School of Food Science and Technology, Dalian Polytechnic University, Dalian 116034, People's Republic of China*

* Corresponding authors:

Xinping Lin, *National Engineering Research Center of Seafood, Collaborative Innovation Center of Provincial and ministerial co-construction for Seafood Deep Processing, Liaoning Province Collaborative Innovation Center for Marine Food Deep Processing，School of Food Science and Technology, Dalian Polytechnic University, Dalian 116034, People's Republic of China.*

Tel.: +86041186318675; fax: +86041186318655. E-mail: yingchaer@163.com

Table S1 Alpha diversity indices of bacteria in *Suanzhayu* with and without lipase.

Sample	OTUs	ACE	Chao1	Simpson	Shannon	Coverage (%)	Sample	OTUs
0d	220	220.61	220.17	0.05	4.08	99.99	0d	220
U0-3d	172	230.59	233.88	0.16	2.36	99.87	U0-3d	172
U0-5d	145	177.84	171.25	0.13	2.53	99.91	U0-5d	145
U0-7d	157	195.03	186.25	0.11	2.67	99.91	U0-7d	157
U0-14d	270	274.42	276.88	0.02	4.65	99.97	U0-14d	270
U100-3d	202	239.78	239.28	0.17	2.35	99.96	U100-3d	202
U100-5d	224	256.09	277.26	0.24	2.08	99.96	U100-5d	224
U100-7d	252	274.19	283.20	0.14	2.45	99.97	U100-7d	252
U100-14d	255	286.77	300.04	0.12	2.65	99.96	U100-14d	255

Table S2 Concentration of volatile compounds in *Suanzhayu* with or without lipase.

Volatile compounds	RT	Content (μg/ kg)								
		0d	U0-3d	U0-5d	U0-7d	U0-14d	U100-3d	U100-5d	U100-7d	U100-14d
Aldehyde (7)										
pentanal	699	319.8±28.6	122.7±17.7	184.3±0.4	586.6±10.5	437.2±3.6	170.2±31.0	288.0±1.0	229.0±1.7	671.2±71.0
hexanal	800	7394.9±1173.7	8152.8±124.5	7305.1±277.5	10729.2±450.6	4992.6±27.0	10583.7±692.2	12868.2±344.4	7536.0±981.3	6931.7±303.5
heptanal	901	148.9±30.1	245.8±12.8	238.6±14.3	376.0±24.1	319.3±47.8	298.5±0.9	423.3±26.1	398.1±42.8	359.4±28.8
benzaldehyde	962	ND	49.3±2.5	182.4±6.4	207.4±17.3	230.8±11.9	71.2±17.6	224.1±4.9	237.5±82.1	253.5±1.0
(E)-2-heptenal	958	ND	162.2±1.2	145.4±2.1	167.9±0.6	243.4±8.0	169.4±9.1	198.1±12.5	227.4±13.2	215.6±16.1
octanal	1003	146.3±28.1	270.0±24.1	443.4±50.0	489.7±1.9	387.0±20.4	319.6±8.5	502.7±14.3	641.6±67.7	839.3±11.4
nonanal	1104	200.2±25.5	434.5±16.8	328.0±51.6	473.8±91.5	391.7±24.2	713.9±85.1	559.6±31.8	551.9±93.4	300.1±2.6
subtotal		8210.0±1286.0	9437.9±136.2	8827.1±372.9	13030.7±574.3	7002.0±119.7	12326.4±824.4	15064.1±288.5	9821.4±1114.6	9570.8±255.0
Alcohols (6)										
3-methyl-1-butanol	736	ND	65.4±3.2	177.1±5.8	107.6±4.8	546.2±64.7	48.1±7.5	1014.8±134.8	1961.4±11.7	1903.9±104.0
1-pentanol	765	ND	117.9±9.1	228.7±16.9	308.2±21.8	303.6±9.5	118.4±2.9	186.3±14.6	180.3±23.0	259.2±67.1

1-hexanol	868	98.5±26.8	699.7±113.5	1881.7±51.3	2731.1±125.9	3938.3±238.8	413.5±290.7	1942.1±29.2	3022.5±153.2	2795.9±31.8
1-octene-3-alcohol	980	624.8±171.8	1853.4±108.5	1356.7±5.2	2067.6±337.1	1829.7±27.7	2229.0±7.8	2926.8±15.2	2020.2±113.2	1794.1±53.1
2,4-dimethyl-cyclohexanol	1032	ND	65.9±1.3	41.0±10.1	63.6±16.6	130.0±2.2	59.6±8.7	101.0±8.5	93.3±15.1	86.8±11.6
(Z)-5-octen-1-alcohol	1056	ND	64.2±2.5	32.8±6.2	43.0±11.6	110.0±21.4	62.6±1.6	65.7±0.7	46.7±8.7	31.0±1.0
Subtotal		723.3±198.7	2866.5±226.8	3718.0±64.9	5321.1±222.4	6857.8±247.2	2931.2±301.0	6236.6±155.6	7324.4±324.9	6870.9±134.3
Ester (2)										
ethyl ester hexanoic acid	1000	ND	ND	28.9±4.8	33.5±3.2	24.7±4.7	ND	21.4±2.9	79.5±4.6	199.9±0.0
ethyl ester octanoic acid	1196	ND	ND	ND	ND	ND	ND	6.5±1.0	12.4±0.1	76.2±0.8
Subtotal		ND	ND	28.9±4.8	33.5±3.2	24.7±4.7	ND	27.9±2.0	91.8±4.5	276.1±0.8
Others (6)										
2,3-octanedione	984	300.3±63.7	93.5±29.5	457.8±60.1	707.1±41.0	396.1±26.5	84.0±6.6	393.1±73.9	658.6±103.0	415.7±0.0

2-pentyl-furan	993	67.6±6.7	141.9±19.2	408.8±83.7	614.7±138.5	447.9±60.4	316.6±133.0	374.2±13.4	869.5±104.7	438.3±5.9
ethyl-pyrazine	921	ND	91.9±4.4	246.3±31.9	224.9±33.4	163.1±21.0	ND	202.4±8.7	242.5±65.9	216.1±12.0
2,3-dimethyl-pyrazine	925	ND	50.1±5.1	194.3±11.9	214.2±19.6	90.2±0.8	ND	95.5±8.6	87.2±30.9	101.7±31.7
2-ethyl-3-methyl-pyrazine	1004	16.7±3.7	95.7±10.3	314.0±0.6	115.6±7.5	377.6±6.5	38.0±6.4	142.0±17.6	209.1±33.0	217.1±43.3
2-ethyl-3,5-dimethyl-pyrazine	1084	9.4±1.1	29.4±4.0	108.1±6.5	53.1±3.8	69.1±9.4	11.7±2.5	56.6±3.0	80.5±5.5	90.1±5.2
Subtotal		394.0±75.2	502.5±26.0	1729.3±130.9	1929.6±228.7	1544.1±139.1	450.4±130.7	1263.7±84.1	2147.3±343.0	1479.0±10.6
Total		9327.3±1559.8	12807.0±389.0	14303.4±573.6	20314.9±564.8	15428.5±16.4	15708.0±654.1	22592.3±46.8	19384.9±1778.0	18196.9±183.1

Values are represented as means ± SD (standard deviation).

ND represents not detected.

RI represents retention indices.

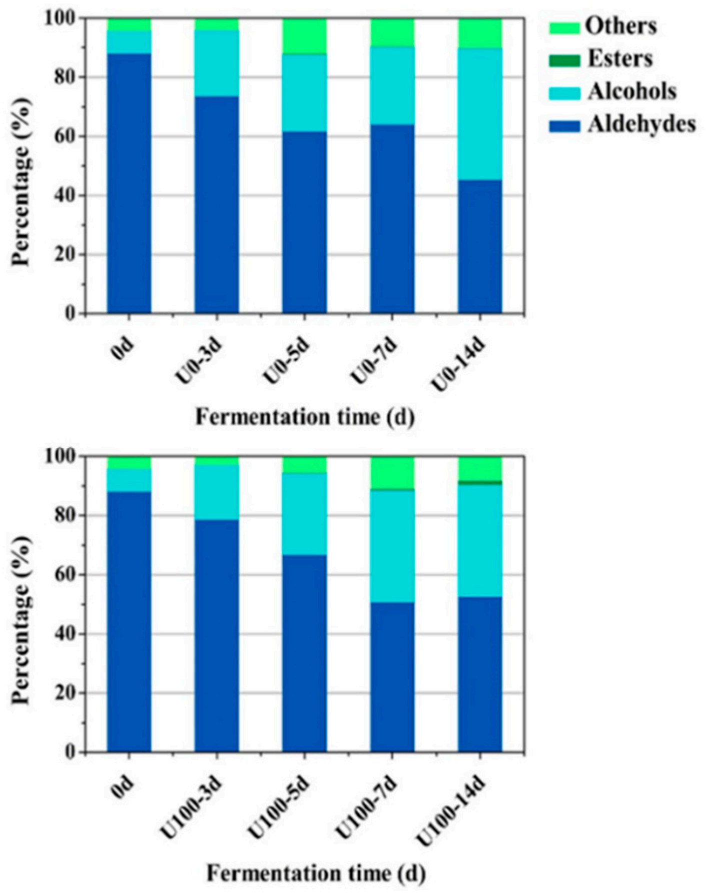

Figure S1 The percentage of volatile compounds during the fermentation *Suanzhayu* samples without (U0) and with (U100) lipase.
